# The relationship between the characteristics of burst suppression pattern and different etiologies in epilepsy

**DOI:** 10.1038/s41598-021-95040-4

**Published:** 2021-08-05

**Authors:** Haipo Yang, Pan Gong, Xianru Jiao, Qiujun Zhou, Yuehua Zhang, Yuwu Jiang, Zhixian Yang

**Affiliations:** Department of Pediatrics, Peking University First Hospital, No. 1, Xi’anmen Street, Xicheng District, Beijing, 100034 China

**Keywords:** Diseases of the nervous system, Encephalopathy, Epilepsy

## Abstract

To analyze the relationship between the characteristics of burst suppression (BS) pattern and different etiologies in epilepsy. Patients with a BS pattern who were younger than 6 months old were screened from our electroencephalogram (EEG) database. The synchronized and symmetric BS patterns under different etiologies in epilepsy were analyzed. A total of 32 patients had a BS pattern on EEG. The etiologies included genetic disorders (37.5%), cortical malformations (28.1%), inborn errors of metabolism (12.5%), and unknown (21.9%). Twenty-five patients were diagnosed with Ohtahara syndrome, one as early myoclonic encephalopathy, and one as epilepsy of infancy with migrating focal seizure. Five cases could not be classified into any epileptic syndrome. Asynchronous BS pattern was identified in 18 cases, of which 13 (72%) patients had genetic and/or metabolic etiologies. Synchronous BS pattern was identified in 14 cases, of which 8 (57%) patients had structural etiologies. Twenty-three patients had symmetric BS patterns, of which 15 (65%) patients had genetic etiologies. Nine patients had asymmetric BS patterns, of which 8 (89%) patients had structural etiologies. Patients with genetic epilepsies tended to have asynchronous and symmetric BS patterns, whereas those with structural epilepsies were more likely to have synchronous and asymmetric BS patterns.

## Introduction

Burst suppression (BS) is an abnormal electroencephalogram (EEG) pattern characterized by the alternating appearance of depressed background activity and bursts of mixed-frequency paroxysmal activity^1^. BS pattern is typically recorded in patients with early-onset epileptic encephalopathy (EOEE), such as Ohtahara syndrome (OS) and early myoclonic encephalopathy (EME)^2–4^. The BS pattern in OS is characterized by high-voltage bursts alternating with nearly flat periods at an approximately regular rate^2^. Bursts of 1- to 3-s duration comprise 150- to 350-microvolt high-voltage slow waves intermixed with multifocal spikes^2^. The duration of the suppression phase ranges from 2 to 5 s. Burst-to-burst interval usually ranges from 5 to10 seconds. The SB pattern of EME becomes more distinct during sleep, especially during deep sleep. The suppression phase is longer in the earlier stage of the disorder. It also tends to become longer in deeper sleep in the same tracing^2^. A few studies mentioned that the BS pattern could also be observed in patients with epilepsy of infancy with migrating focal seizures (EIMFS)^5,6^. Asymmetric BS pattern was reported in about two-thirds of cases, but no remarkable asynchronization was found except in the case of Aicardi’s syndrome^7^. Asynchronous and asymmetric BS pattern could be seen in patients with corpus callosum lesions^8^. Asymmetric BS pattern was usually observed in hemimegaloencephaly^9,10^. Studies showed that the synchronization and symmetry of BS pattern was related to different epileptic syndromes^2,7,10^. For example, the BS pattern in OS was bilaterally synchronous but some asymmetry between interhemispheres^7,10^. In EME, the BS pattern was often asynchronous and had irregular burst-to-burst intervals^2,11^. As for EIMFS, BS could be synchronous or asynchronous^6^. In this study, we mainly aimed to analyze the relationship between the characteristics of BS patterns and different etiologies in epileptic syndromes or epilepsy.

## Patients and methods

### Patients

**Figure 1:**
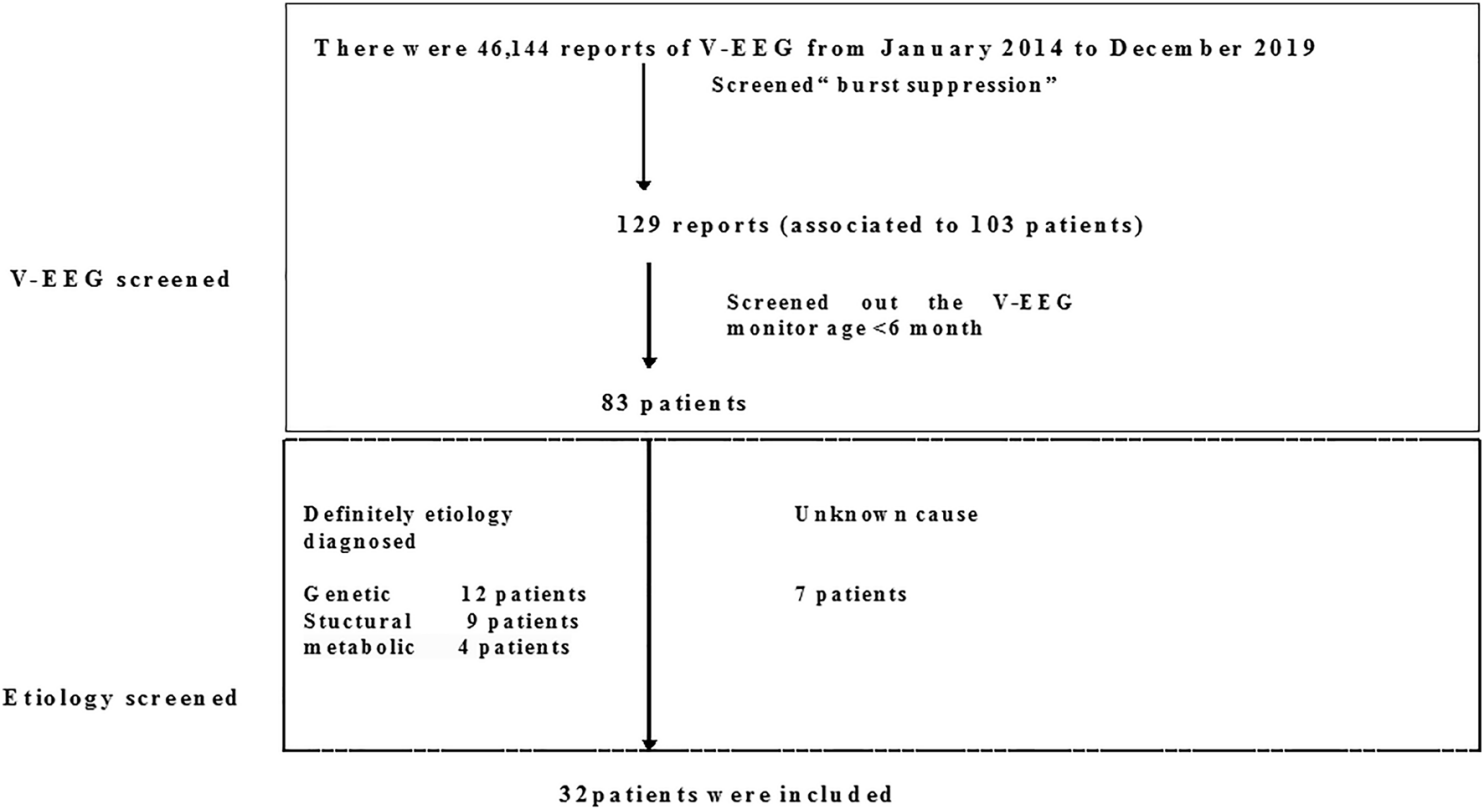
The flow diagram of patient's screening result.

A total of 46,144 video-EEG (VEEG) recordings were screened from January 2014 to December 2019 at the Department of the Pediatrics of Peking University First Hospital. Firstly, a total of 129 EEG recordings were screened out from all the EEG data using the keywords “burst suppression pattern”. Secondly, these EEG recordings were associated with 103 patients (we will select the first EEG recording if one patient has more than one EEG recordings), among whom 83 patients younger than 6 months old at the time of the VEEG monitoring were chosen. Thirdly, patients who were diagnosed with genetic, structural and metabolic etiologies were selected. Patients with unknown causes were also included, provided that they must have completed genetic testing, brain magnetic resonance imaging (MRI) scanning, and metabolic workup with negative results. Finally, a total of 32 patients were included in this study (Fig. 1).

The following demographic data were analyzed: gender, age at seizure onset, perinatal and personal history, and relevant data. Genetic testing was performed by whole-exome sequencing (WES) or gene panel. For patients who had undergone gene panel test, targeted sequencing was performed on genomic DNA samples from the proband and his/her parents for 535 epilepsy genes (supplementary table 1) by MyGenostics Technology, Inc. (Beijing, China). The etiologies were classified according to the etiological classification guidelines revised by the International League Against Epilepsy (ILAE) in 2017, and the epileptic syndromes were classified according to the classification revised by ILAE in 2010^12,13^.

### VEEG monitoring

EEG was recorded using the standard international 10–20 system, with a sampling frequency of 500 Hz (Neurofax; Nihon-Kohden, Tokyo, Japan). A low-cut filter at 1.6 Hz was used before digital sampling. A 4-h VEEG monitoring was performed. All cases must complete 4-h surveillance, including at least one complete wake-sleep cycle. All EEGs were analyzed by one EEG technician and at least two certified epileptologists in order to reach consensus conclusions. According to Andre M, et al.^14^, asynchrony implied a temporal delay longer than 1.5 to 2 s between the bursts of identical waveforms on both hemispheres, and the amplitude difference exceeding 50% over one hemisphere was considered as an abnormal EEG. We, therefore, defined interhemispheric synchrony as that a temporal delay was no longer than 1.5 to 2 s between the bursts of identical waveforms between hemispheres, and bilateral hemispheric asymmetry as that the amplitude of BS pattern in one hemisphere was 50% higher than the other.

### Statistical analysis

For comparing the VEEG monitor age between the synchronous and the asynchronous group, an independent-sample *t*-test was performed using SPSS 16. The chi-square test was used to analyze the relationship between different patterns and etiologies. Results were considered statistically significant at *p* < 0.05.

This study was approved by the Ethical Committee of Peking University First Hospital, approval number: 2016-1135. Written informed consent was obtained from the legal guardians (parents) of the patient. All methods were carried out in accordance with relevant guidelines and regulations..

## Results

### General information

A total of 32 patients (20 boys and 12 girls) were involved in this study. One was born at pre-term (34 weeks) and the remainders were born at term. Six patients had abnormal birth history, including fetal heart rate monitor abnormality in two, premature rupture of membranes and amniotic fluid pollution in one, amniotic fluid pollution in one, malposition of the fetus and anoxia in one, threatened abortion in one. The median age of seizure onset was 3 days (range from 1 to 90 days). The median age of the patients who underwent EEG examination was 58.5 days (range from 19 to 168 days).

### The relationship between etiologies and epileptic syndromes or epilepsy (Table 1)

**Table 1:** The etiologies of patients.

Etiology	Gene test			Brain MRI			Metabolism screening			Diagnosed			
	Normal	Abnormal	Not done	Normal	Cortical malformations	Nonspecific abnormal	Normal	Abnormal	Not done	OS	EME	EIMFS	EP
Genetic (n)	0	12	0	10	0	2	9	0	3	11	0	1	0
Structural (n)	1	0	8	0	9	0	6	0	3	8	0	0	1
Metabolic (n)	0	2	2	0	0	4	0	4	0	0	0	0	4
Unknown (n)	7	0	0	3	0	4	7	0	0	6	1	0	0
%	25.0%	43.8%	31.2%	40.6%	28.1%	31.3%	68.8%	12.5%	18.7%	78.1%	3.1%	3.1%	15.6%

*OS* Ohtahara syndrome, *EME* Early myoclonic encephalopathy, *EIMFS* Epilepsy of infancy with migrating focal seizure, *EP* Epilepsy, *MRI* Magnetic resonance imaging.

Of the 32 patients, 25 patients were diagnosed as OS, one as EME, and one as EIMFS. Five cases could not be classified into any epileptic syndrome.

Thirty-two patients were divided into the following categories based on their etiologies: 12 patients (12/32, 37.5%) had genetic disorders, including eight patients who underwent WES and four patients who underwent the gene panel. The pathogenic variants were identified in the following genes (Table 2): *KCNQ2* in five cases, *SCN2A* in four, *KCNT1* in one, *STXBP1* in one, and *GNAO1* in one. Two out of the 12 patients had nonspecifically abnormal findings on brain MRI, including the abnormal signal of the patchy center of left hemioval and dysplasia of the corpus callosum. Eleven patients were diagnosed with OS and one with EIMFS (caused by a mutation in *KCNT1*). Cortical malformations were found in nine patients (9/32, 28.1%), including focal cortical dysplasia in four and hemimegalencephaly in five. In these nine patients, eight were diagnosed with OS; one could not be diagnosed with any epileptic syndrome. Four patients (4/32, 12.5%) had metabolic abnormalities, including methylmalonicacidemia (MMA) in three, and carnitine-acylcarnitine translocase deficiency (CACTD) in one. All these four patients had brain MRI abnormalities, including the ventricular system expansion and hydrocephalus in three MMA patients, and bilateral semioval center, lateral ventricle, an abnormal signal of white matter in one CACTD patient. One out of three patients with MMA was confirmed carrying homozygous mutations in the metabolism of cobalamin-associated C (MMACHC). The CACTD patient was confirmed carrying compound heterozygous mutations in *SLC25A20*. Seven cases (7 /32, 21.9%) were classified into the group with unknown causes. Four out of them had abnormal brain MRI findings, including a small number of abnormal signals in the posterior ventricle angle in one patient, delayed myelination of the white matter and widened subarachnoid space of the bilateral front temporal lobes in one, corpus callosum thin in one and extra encephalic space widened in one case. In the seven patients, six were diagnosed with OS, one was categorized into EME.

**Table 2:** The gene mutation types of patients.

ID	Epilepsy gene	Suspected or known pathogenic mutation	Inheritance pattern	Parental origin	Inheritance type	Phenotype
1	KCNQ2	c.736G > C	Heterozygous	De novo	AD	OS
2	KCNQ2	c.647C > T	Heterozygous	De novo	AD	OS
3	KCNQ2	c.740C > T	Heterozygous	De novo	AD	OS
4	KCNQ2	c.829A > G	Heterozygous	De novo	AD	OS
5	KCNQ2	c.794C > T	Heterozygous	De novo	AD	OS
6	SCN2A	c.1108 T > C	Heterozygous	De novo	AD	OS
7	SCN2A	c.655 T > G	Heterozygous	De novo	AD	OS
8	SCN2A	c.468G > T	Heterozygous	De novo	AD	OS
9	SCN2A	c.2604 T > A	Heterozygous	De novo	AD	OS
10	STXBP1	c.143-144insA	Heterozygous	De novo	AD	OS
11	GNAO1	c.810C > A	Heterozygous	De novo	AD	OS
12	KCNT1	c.1420C > T	Heterozygous	De novo	AD	EIMFS
13	SLC25A20	c.842C > T;c.199-10 T > C	Compound heterozygous	Paternal; Maternal	AR	CACTD
14	MMACHC	c.609G > A; c.609G > A	Homozygous	Paternal; Maternal	AR	MMA

*OS* Ohtahara syndrome, *EIMFS* Epilepsy of infancy with migrating focal seizure, *CACTD* Carnitine-acylcarnitine translocase deficiency, *MMA* Methylmalonic academia, *AD* Autosomal dominant, *AR* Autosomal recessive.

### The relationship between EEG features and epileptic characteristics (Table 3)

**Table 3:** The relationship between EEG features and epileptic characteristics.

BS pattern	Etiology				Chi-square test	Epileptic syndrome and Epilepsy			
	Genetic	metabolic	Structural	Unknown		OS	EME	EIMFS	EP
Synchronous	3	0	8	3	*P* = 0.006	12	1	0	1
Asynchronous	9	4	1	4		13	0	1	4
Symmetrical	11	4	1	7	*P* = 0.000	17	1	1	4
Asymmetrical	1	0	8	0		8	0	0	1

*BS* Burst suppression, *OS* Ohtahara syndrome, *EME* Early myoclonic encephalopathy, *EIMFS* Epilepsy of infancy with migrating focal seizure, *EP* Epilepsy.

**Figure 2:**
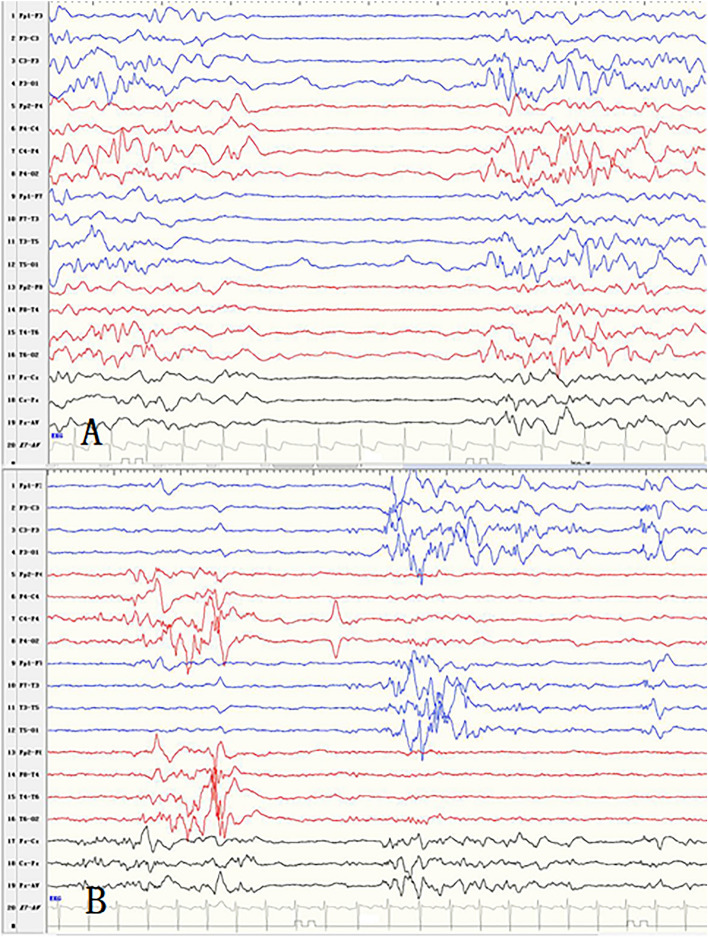
(**A**) The synchronous and symmetric burst suppression pattern in a patient with genetic disorder. (**B**) The asynchronous but symmetric burst suppression pattern in a patient with genetic disorder.

Interhemispheric synchrony in BS pattern was observed in 14 patients, including three cases of genetic epilepsy, eight of structural epilepsy, and three of epilepsy with unknown causes (Fig. 2). Twelve patients were diagnosed with OS, one with EME, and one could not be diagnosed with any epileptic syndrome. Interhemispheric asynchrony in BS pattern was observed in 18 patients, including nine cases of genetic epilepsy, one of structural epilepsy, four of metabolic epilepsy, and four of epilepsy with unknown causes. Of these 18 patients, 13 patients were diagnosed with OS, one with EIMFS, and four patients could not be diagnosed with any epileptic syndrome. The relationship between synchronism and the etiologies was analyzed by the Chi-square test. The results showed significant differences among different etiologies with synchronous and asynchronous BS patterns (*p* = 0.006). The median ages of EEG detection in the synchronous group and asynchronous group were 58.5 days and 55 days , respectively. The difference was not statistically significant by independent *t*-test analysis using SPSS (*p* = 0.40).

**Figure 3:**
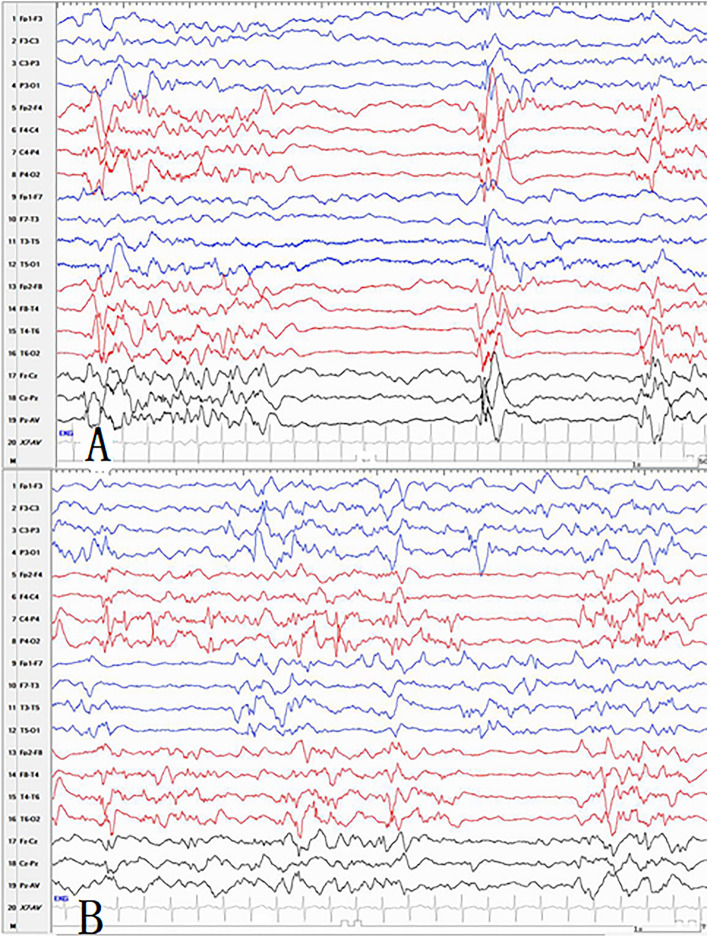
(**A**) The synchronous and asymmetric burst suppression pattern in a patient with structural etiology. (**B**) The asynchronous but asymmetric burst suppression pattern in a patient with genetic disorder.

Twenty-three out of the 32 patients (71.9%) had interhemispheric symmetry BS patterns, including 11 patients of genetic epilepsy, one of structural epilepsy, four of metabolic epilepsy, and seven of epilepsy with unknown causes (Fig. 3). In the 23 patients, 17 were diagnosed as OS, one as EME, one as EIMFS, and the remaining four patients could not be diagnosed with any epileptic syndrome. Nine out of all patients (28.1%) showed asymmetry BS pattern, including one patient with genetic epilepsy and eight with structural epilepsy. Further, eight patients were diagnosed as OS, and one patient could not be diagnosed with any epileptic syndrome. There were significant differences between different etiologies with symmetric and asymmetric BS pattern analyzed by the Chi-square test. (*p* = 0.000).

## Discussion

In the present study, 37.5% of patients had genetic disorders, 28.1% patients had cortical malformations, 12.5% patients had metabolism abnormalities, and 21.9% patients had unknown causes. Previous studies showed that the proportion of genetic etiologies in BS pattern children was 20–30%^15–17^. Besides 12 patients with genetic disorders, the present study also involved four patients with metabolism abnormalities with definite or possible genetic etiologies. Therefore, the proportion of genetic disorders in our study was about 50% (16/32), which was higher than the previous reports^15–17^. In the study of Olsen et al.^18^, they identified pathogenic variants in 61% of patients excluding those with cortical malformations, and the most commonly identified gene was *KCNQ2*. In our study, after excluding the nine patients with hemimegalencephaly or focal cortical dysplasia, the percentage of patients with genetic disorders was further up to 69.6% (16/23). Consistent with the previous study, *KCNQ2* was also the most common gene^18^.

In the present study, 14 patients had synchronous BS patterns, eight of whom had cortical malformations, suggesting that the BS pattern in patients with structural etiologies tended to be synchronous. Eighteen patients had asynchronous BS patterns, and 13 out of them had genetic disorders (including four patients with metabolic abnormality), which suggested that the BS patterns in patients with genetic etiologies tended to be asynchronous. The statistical analysis also confirmed that there was a significant difference between different etiologies under synchronous and asynchronous BS patterns. Twenty-three patients had symmetric BS patterns, of whom 15 patients had genetic disorders (including four patients with metabolic abnormality), indicating that the BS pattern in patients with genetic etiologies tended to be symmetric. Asymmetric BS pattern predominantly appeared in the malformed hemisphere in hemimegalencephaly, suggesting that the brain lesion extended diffusely from the subcortical layer to cortex^19,20^. In our cohort, nine patients had asymmetric BS patterns, and 88.9% of patients had cortical malformations. In the asymmetric BS pattern, the location of prominent discharges was in accordant with the cortical malformations side, which was consistent with the previous reports^19,20^. The statistical analysis also confirmed that there was a significant difference between different etiologies under symmetric and asymmetric BS patterns.

OS was usually caused by obvious static brain lesions, such as brain malformations even in the early stages^7,19,21^. The BS pattern in OS was reported typically and bilaterally synchronous between hemispheres^10^, but might show some asymmetry^7^. Recently, many genes have been found in patients with OS, such as *KCNQ2*, *SCN2A*, *STXBP1,* and so on^22^. In the present study, 44% of OS patients had genetic abnormalities, while 32% of OS patients had brain MRI abnormalities. For the 25 OS patients, the synchronization and symmetry analysis showed that it presented with asynchronous and symmetrical BS pattern in 48% of patients, synchronous and asymmetrical BS pattern in 28%, synchronous and symmetrical BS pattern in 20%, and asynchronous and asymmetrical BS pattern in 4%. Therefore, patients with OS could have different forms of BS pattern, which might be related to the different etiologies with prominent genetic etiology.

Previous literature showed that EME was most often caused by inherited metabolic disorders, such as nonketotic hyperglycinemia, organic acidemias, or mitochondrial cytopathies^23^. The BS pattern often showed some asynchrony, irregular or longer burst-to-burst intervals, or more multifocal spikes in the suppression phase^2^. As for the present study, the etiology of one case with EME was unknown. The BS pattern of this patient was synchronous and symmetrical, which was different from the previous report^2^. Thus, the BS pattern of EME should also be considered to be closely related to different etiologies.

Genetic causes were found in 69% of patients with EIMFS, and the most commonly involved gene was *KCNT1*^24^. BS was divided into two patterns in patients with EIMFS, one was an early-onset asynchronous pattern, and the other was a late-onset synchronous pattern^6^. In our study, six patients were diagnosed with EIMFS, of whom four patients had *KCNT1* mutations, two patients did not undergo genetic tests^6^. In the four patients with *KCNT1* mutation, BS in one patient belonged to the early-onset pattern, and BS in three patients belonged to the late-onset pattern^6^. The age at EEG recording of the early-onset pattern was within one month after birth, and the asynchronous BS pattern might be associated with premature brain development^6^, because the degree of synchrony might approach 100% in normal-term neonates^1^. Inconsistent with previous studies, our patient with EIMFS caused by *KCNT1* mutation had an asynchronous BS pattern, and the age at EEG recording was 2 months. In order to observe the influence of the developing brain maturity on the BS pattern, the EEG examination age of the synchronous group and asynchronous group was compared, and there was no significant difference in the EEG examination age between the two groups. Hence, the premature EEG could not fully explain the asynchronous BS pattern, and the etiologies might also play a role.

Corpus callosum immature or dysfunction was reported to be associated with the asynchronous and symmetry of BS pattern^8,25^. The association of asynchronous BS with Aicardi's syndrome supported the role of the corpus callosum in interhemispheric synchrony^26^. Two out of our patients with asynchronous BS patterns had corpus callosum abnormal, which was consistent with the previous report^26^. However, another literature reported that children with normal corpus callosum also had asynchrony EEG pattern^5^. The persistence of bilaterally synchronous interictal EEG discharges following partial or complete corpus callosum section suggested that corpus callosum was not the sole pathway for hemisphere synchronization^27^. Experimental evidence suggested that a pathway between ipsilateral and contralateral cortex relaying via thalamic and mesencephalic subcortical structures might contribute to secondary bilateral synchrony. Abnormal thalamic loop or abnormal synaptic development was associated with bilateral hemispheric asynchrony as well^27,28^. Indeed, 16 out of our patients with asynchronous BS patterns had normal corpus callosum, and of whom 11 patients had genetic disorders. Therefore, the asynchronous BS pattern might be associated with genetic etiologies.

## Conclusion

As this was a retrospective study and the patients were retrieved from the EEG database, we could only analyze the limited and available EEG data. BS pattern could be observed in a variety of epilepsy syndromes. The most common epileptic syndrome was OS in our study. Genetic etiology was commonly identified. Asynchronous and symmetrical BS patterns were mostly observed in patients with genetic etiologies. Whereas, synchronized but asymmetrical BS patterns were found in patients with structural etiologies. Our findings suggested that characteristics of the BS patterns could help to predict the etiology of the patient with epilepsy.

## Supplementary Information

Supplementary Information.
