# Supplementary material for: The relationship between the characteristics of burst suppression pattern and different etiologies in epilepsy
Source: Sci Rep. 2021 Aug 5;11:15903. doi: 10.1038/s41598-021-95040-4 (PMC8342459; doi:10.1038/s41598-021-95040-4)
Supplement: Supplementary file 1 — Supplementary Information. [file 41598_2021_95040_MOESM1_ESM.docx]

[Supplementary](javascript:;) table 1

The 535 genes were included in the panel.

| ABAT | BCKDK | CTSA | FASTKD2 | HNF1B | MDGA2 | OPHN1 | RAB3GAP1 |
| --- | --- | --- | --- | --- | --- | --- | --- |
| ABCC2 | BCS1L | CTSD | FCGR2B | HNRNPH1 | ME2 | PAFAH1B1 | RAF1 |
| ABCC8 | BOLA3 | CTSF | FGD1 | HNRNPU | MECP2 | PAH | RANBP2 |
| ACADSB | BRAF | CUL4B | FGF8 | HP | MED12 | PAK3 | RARS2 |
| ACOX1 | BSN | CYB5R3 | FGFR3 | HPD | MEF2C | PANK2 | RBFOX1 |
| ACTB | BTD | CYP2R1 | FH | HRAS | MFSD8 | PAX6 | RBFOX2 |
| ACY1 | C10orf2 | CYP2U1 | FKRP | HSD17B10 | MGAT2 | PC | RBFOX3 |
| ADCK3 | C12orf65 | D2HGDH | FKTN | HSD17B4 | MLC1 | PCDH19 | RELN |
| ADK | C4A | DAO | FLNA | HTR2A | MMACHC | PCNT | RFT1 |
| ADSL | CACNA1A | DAOA | FOLR1 | HTT | MOCS1 | PDHA1 | RNASEH2A |
| AFG3L2 | CACNA1H | DBH | FOXG1 | HYAL1 | MOCS2 | PDHX | RNASEH2B |
| AGA | CACNB4 | DBT | FOXP2 | IDH2 | MOCS3 | PDSS1 | RNASEH2C |
| AHI1 | CACNG2 | DCX | FOXRED1 | IDS | MOGS | PDSS2 | ROGDI |
| AKT1 | CASK | DDC | FUCA1 | IDUA | MPC1 | PEX1 | RPGRIP1L |
| ALDH4A1 | CASR | DDOST | GABBR2 | IER3IP1 | MPDU1 | PEX10 | RPIA |
| ALDH5A1 | CC2D2A | DEPDC5 | GABRA1 | IFNG | MPI | PEX12 | RTN4R |
| ALDH7A1 | CDH13 | DGKD | GABRA6 | IL6 | MR1 | PEX13 | RYR1 |
| ALG1 | CDH9 | DHCR7 | GABRB2 | INPP5E | MTHFR | PEX14 | RYR3 |
| ALG11 | CDKL5 | DHFR | GABRB3 | INS | MTOR | PEX16 | SAMHD1 |
| ALG12 | CEP152 | DIAPH3 | GABRD | IQSEC2 | MTR | PEX19 | SCARB2 |
| ALG13 | CEP290 | DISC1 | GABRG2 | KCNA1 | MTRR | PEX2 | SCN1A |
| ALG2 | CHD2 | DLD | GALC | KCNB1 | NAGLU | PEX26 | SCN1B |
| ALG3 | CHI3L1 | DMPK | GALNS | KCNH5 | NDE1 | PEX3 | SCN2A |
| ALG6 | CHRNA2 | DNAJC5 | GAMT | KCNJ1 | NDN | PEX5 | SCN4A |
| ALG8 | CHRNA3 | DNAJC6 | GATM | KCNJ10 | NDUFA1 | PEX6 | SCN8A |
| ALG9 | CHRNA4 | DNASE1 | GBA | KCNJ11 | NDUFA11 | PEX7 | SCN9A |
| AMACR | CHRNA5 | DNM1 | GCDH | KCNMA1 | NDUFA2 | PGK1 | SCO2 |
| AMER1 | CHRNA7 | DOCK6 | GCK | KCNQ1 | NDUFAF1 | PGM1 | SDHA |
| AMT | CHRNB2 | DOCK7 | GCSH | KCNQ2 | NDUFAF2 | PHF6 | SERPINI1 |
| APOL2 | CLCN2 | DOLK | GFAP | KCNQ3 | NDUFAF3 | PHGDH | SETBP1 |
| APOL4 | CLCN4 | DPAGT1 | GLB1 | KCNT1 | NDUFAF4 | PIGA | SGCE |
| APP | CLCNKA | DPM1 | GLDC | KCTD7 | NDUFAF5 | PIGL | SGSH |
| APTX | CLCNKB | DPM3 | GLRA1 | KDM5C | NDUFB3 | PIGV | SHANK3 |
| ARG1 | CLN3 | DPYD | GLRB | KIF11 | NDUFS1 | PLA2G6 | SHH |
| ARHGAP31 | CLN5 | DRD2 | GLUD1 | KIF1A | NDUFS2 | PLCB1 | SHOC2 |
| ARHGEF15 | CLN6 | DRD3 | GLUL | KMT2D | NDUFS3 | PLP1 | SIX3 |
| ARHGEF9 | CLN8 | DTNBP1 | GNE | KRAS | NDUFS4 | PMM2 | SLC13A5 |
| ARL13B | CNTN5 | EBP | GNPTAB | KRIT1 | NDUFS6 | PNKD | SLC16A2 |
| ARSA | CNTNAP2 | ECM1 | GNPTG | L2HGDH | NDUFS7 | PNKP | SLC17A5 |
| ARSB | COA5 | EEF1A2 | GNS | LAMA2 | NDUFS8 | PNPO | SLC19A3 |
| ARSE | COG1 | EFHC1 | GOSR2 | LARGE | NDUFV1 | POLG | SLC1A3 |
| ARX | COG4 | EHMT1 | GPC3 | LBR | NDUFV2 | POMGNT1 | SLC20A2 |
| ASAH1 | COG5 | EIF2B1 | GPHN | LGI1 | NEDD4L | POMT1 | SLC25A15 |
| ASPA | COG6 | EIF2B2 | GPR56 | LGR4 | NEU1 | POMT2 | SLC25A19 |
| ATIC | COG7 | EIF2B3 | GPR98 | LIAS | NF1 | PPOX | SLC25A22 |
| ATN1 | COG8 | EIF2B4 | GRIA3 | LIG4 | NGLY1 | PPT1 | SLC26A4 |
| ATP13A4 | COL18A1 | EIF2B5 | GRIN1 | LMX1B | NHLRC1 | PQBP1 | SLC2A1 |
| ATP1A2 | COL4A1 | ELP4 | GRIN2A | LRPPRC | NHS | PRICKLE1 | SLC35A1 |
| ATP1A3 | COMT | EMX2 | GRIN2B | MAGI1 | NID2 | PRICKLE2 | SLC35A2 |
| ATP2A2 | COQ2 | EPB41L1 | GSS | MAGI2 | NOTCH3 | PROC | SLC35C1 |
| ATP5A1 | COQ9 | EPHB2 | GUSB | MAGT1 | NPC1 | PRODH | SLC46A1 |
| ATP6AP2 | COX14 | EPM2A | GYS1 | MAN1B1 | NPC2 | PRRT2 | SLC6A8 |
| ATP7A | COX15 | ERBB4 | HAX1 | MANBA | NPHP1 | PSAP | SLC9A6 |
| ATPAF2 | COX6B1 | ERLIN2 | HDAC4 | MAP2K1 | NR3C1 | PSAT1 | SLC9A9 |
| ATRX | CPA6 | ETFA | HEXA | MAP2K2 | NRAS | PTCH1 | SMC1A |
| ATXN10 | CPS1 | ETFB | HEXB | MAPK10 | NRXN1 | PTPN11 | SMPD1 |
| B4GALT1 | CPT1A | ETFDH | HFE | MBD5 | NTNG1 | PTPN22 | SMS |
| BANK1 | CPT2 | EVC | HGSNAT | MCCC2 | NUBPL | PUS1 | SNIP1 |
| BCKDHA | CSTB | FADD | HLA-DQA1 | MCOLN1 | OFD1 | QDPR | SNRPN |
| BCKDHB | CTNNA3 | FASN | HLA-DQB1 | MCPH1 | OPA1 | RAB39B | SOBP |
| SOS1 | SYNGAP1 | TRPM6 | ZDHHC15 | SUCLA2 | TMEM67 | VPS13A | TUBGCP6 |
| SPAST | SYNJ1 | TSC1 | ZEB2 | SUMF1 | TMEM70 | VPS13B | TUSC3 |
| SPTAN1 | SYP | TSC2 | ZFYVE26 | SUOX | TNK2 | VRK1 | TYROBP |
| SPTLC2 | SZT2 | TSEN2 | ZNF41 | STXBP1 | TMEM216 | UBE3A | ST3GAL5 |
| SRD5A3 | TACO1 | TSEN34 | SURF1 | TPP1 | VRK2 | TBX1 | STRADA |
| SRPX2 | TBC1D24 | TSEN54 | SYN1 | TREM2 | WDR45 | TCF4 | STS |
| ST3GAL2 | TBP | TUBA1A | SYN2 | TREX1 | XK | TMEM165 |  |
